# Creating patient safety capacity in a nation's health system: A comparison between Israel and Canada

**DOI:** 10.1186/2045-4015-1-19

**Published:** 2012-05-23

**Authors:** Roy Ilan, Yoel Donchin

**Affiliations:** 1 Department of Medicine and Critical Care Program, Queen's University, Kingston General Hospital, Etherington Hall, Room 1005, 94 Stuart Street, Kingston, ON, K7L 3N6, Canada; 2 Hadassah Hebrew University Medical School, PO Box 12007, Jerusalem, 91120, Israel

**Keywords:** Safety, Quality Improvement, National Health Policy

## Abstract

Injuries to patients by the healthcare system (i.e., adverse events) are common and their impact on individuals and systems is considerable. Over the last decade, extensive efforts have been made worldwide to improve patient safety. Given the complexity and extent of the activities required to address the issue, coordinating and organizing them at a national level is likely beneficial. Whereas some capacity and expertise already exist in Israel, there is a considerable gap that needs to be filled. In this paper two countries, Canada and Israel, are examined and some of the essential steps for any country are considered. Possible immediate next steps for Israel are suggested.

## Background

In 1966, the National Academy of Sciences published the report "Accidental Death and Disability: The Neglected Disease of Modern Society", [1] demonstrating that soldiers who were seriously wounded on the battlefields of Vietnam had a better survival rate than those individuals who were seriously injured in motor vehicle accidents on California freeways. The response in the U.S. was swift. The Paramedic, a new health care profession, was created; helicopters were introduced to facilitate timely transportation of injured patients to hospitals; and the Emergency Medical Services' response time was reduced. In Baltimore, Dr. R. Adams Cowley founded the Shock Trauma Center, a hospital dedicated to caring for trauma patients; many other trauma centers were established throughout the U.S.

In the last decade of the 20th century, accumulating evidence and sentinel publications focused on adverse events and iatrogenic injury in health care. Anesthesiologists were the first to investigate the huge numbers of mishaps in anesthesia and establish a foundation for improving patient safety. The Australia Health Care Study, [2] "To Err is Human: Building a Safer Health System", [3] and "An Organization with a Memory" [4] revealed that large proportions of hospitalized patients in Australia, the U.S., and the U.K. have suffered an adverse event. A substantial proportion of the adverse events, many of them deemed preventable, resulted in death or severe injury.

Following the publication of these reports, each of the involved countries established high profile committees with a mandate to examine the issue of patient safety and develop recommendations to address system deficiencies. These efforts have included strong support from federal and state governments. In addition, a variety of professional groups, employers, regulators, and healthcare providers have initiated a wide range of efforts to address this issue. Other countries have had a wide range of responses to the need for patient safety. In this paper, we examine two countries, Canada and Israel, and consider some of the essential steps for any country and some possible next steps for Israel. Canadian and Israeli healthcare systems are similar: both are based on public services that are regulated by government. This, in addition to the authors' familiarity with activities in recent years in Canada and Israel, allows for a rich description of the current status in both countries, as well as appropriate recommendations.

## The Canadian Perspective

The Canadian Adverse Events Study, published in 2004, [5] revealed that, similar to other countries, 7.5% of all patients admitted to acute care hospitals in Canada experienced an adverse event. Over a third of all adverse events were judged to be preventable; 20.8% of patients with adverse events died. Although this was the first large-scale report of Canadian data, activities to address patient safety concerns in Canada started prior to 2004.

A series of symposia was held annually in Canada to raise awareness of the problems among healthcare professionals, provide information and training to people working in the new patient safety disciplines, import lessons and tools from other countries and industries, and advance the field of patient safety generally. These annual events, known as the Halifax symposia, ran for ten years, commencing in 2001 and concluding in 2010 [6].

In September 2001, following a one-day forum on patient safety hosted by The Royal College of Physicians and Surgeons of Canada, the National Steering Committee on Patient Safety was first convened. In September 2002, the Steering Committee published *Building a Safer System*, a comprehensive document listing 19 key recommendations for work that must be undertaken within the national integrated strategy [7]. The report suggested five major objectives, including (i) establishing a Canadian patient safety institute to facilitate a national integrated strategy for improving patient safety; (ii) improving legal and regulatory processes; (iii) improving measurement and evaluation processes; (iv) establishing educational and professional development programs; and (v) improving information and communication processes.

Less than a decade later, much progress has been made in all areas identified by the Steering Committee in 2002, as follows:

### A Canadian patient safety institute

The Canadian Patient Safety Institute (CPSI) was established in December 2003 with a mandate from government to build and advance a safer health system for Canadians [8]. CPSI's objectives were to bring innovative solutions to enhance patient safety; facilitate collaboration among governments and stakeholders; support the development of patient safety education programs; provide patients and their families with information and support; and facilitate research in the field.

### Improved legal and regulatory processes

Several legislative efforts have addressed potentially contentious issues related to adverse events. The Apology Act is "... a cultural shift, which recognizes that offering a sincere apology or expression of regret is simply the right thing to do," as described by Phil Hassen, former CEO of the CPSI. The Bill provides that "... an apology made by or on behalf of a person in relation to any civil matter does not constitute an admission of fault or liability by the person... does not affect the insurance coverage available to the person making the apology and is not admissible in any judicial civil proceeding." [9] The first Canadian apology legislation was passed in 2006 by the provinces of British Columbia and Saskatchewan, followed in 2008 by Manitoba. Ontario and Alberta have since introduced similar legislation. The protection afforded by apology laws is similar across Canadian jurisdictions [10]. Similarly, the *Evidence Act *and related legislation within Canadian jurisdictions have been reviewed and revised to ensure that data and opinions associated with patient safety and quality-improvement discussions are protected from disclosure in legal proceedings. In addition, recent activities by regulatory bodies, provincial governments, healthcare providers, professional associations, and others have provided a foundation for the development of pan-Canadian guidelines for disclosure of adverse events to patients. In 2008, the Canadian Disclosure Guidelines were released by the CPSI [11]. From the regulatory perspective, Accreditation Canada, a not-for-profit, independent organization, provides healthcare organizations with an external peer review to assess the quality of their services based on standards of excellence. Accreditation Canada emphasizes health system performance, risk prevention planning, client safety, performance measurement, and governance [12]. As such, many "best practices" and safety procedures are being sustainably enforced by Accreditation Canada. Finally, the *Excellent Care for All Act*, passed into law in Ontario in 2010, is a recent milestone [13]. Among other provisions, this act requires all hospitals in that province to develop an annual plan to improve safety and quality, and links executive compensation to the improvement of care. It reflects the aim of the Ontario government to drive accountability for safety and quality up from front-line healthcare professionals to the executive leaders of organizations. Similar bills are now expected in other Canadian provinces.

### Measurement and evaluation

In 2005, the CPSI introduced to Canada Safer Healthcare Now, [14] a campaign based on the American Institute for Healthcare Improvement's (IHI) 100K Lives Campaign (now 5 million lives campaign), [15] promoting the use of bundles of practices to achieve good clinical outcomes for specific objectives. For example, the first version of the Acute Myocardial Infarction (AMI) bundle, published in 2005, included the following practices: early administration of aspirin; aspirin at discharge; beta-blocker at discharge; timely initiation of reperfusion (thrombolysis or percutaneous intervention); ACE-inhibitor or angiotensin receptor blockers at discharge for patients with systolic dysfunction; and smoking cessation counseling/nicotine replacement/serotonin uptake inhibitor/referral to cardiac rehabilitation program. In 2007, an additional practice, statins at discharge, was added to the bundle. In addition to literature review and educational materials, the campaign provides specific measurement tools for participating organizations to evaluate their performance regarding both processes of care and clinical outcomes. The freely available list of "bundled" interventions and measurement tools promoted by the CPSI is gradually growing and currently includes AMI, medication reconciliation, rapid response teams, and delivery of high-risk medications, as well as measures to prevent nosocomial superbug infections, central line infections, falls, surgical site infections, ventilator associated pneumonia, and venous thromboembolism.

In addition, surveillance systems, including relevant patient-safety indicators, have been developed in Canadian healthcare. The Canadian Institute for Health Information (CIHI) [16] collects and analyzes information on health and healthcare in Canada and makes it publicly available. Canada's federal, provincial, and territorial governments have created CIHI as a not-for-profit, independent organization dedicated to forging a common approach to Canadian health information. CIHI's data and reports, including the Hospital Standardized Mortality Ratio (HSMR), inform health policies, support the effective delivery of health services, and raise awareness among Canadians of the factors that contribute to good health.

Finally, to facilitate progress and improve the evaluation of various interventions in the area, research granting organizations such as the Canadian Institutes for Health Research [17] and the Canadian Health Services Research Foundation [18] have designated competitions dedicated to research in patient safety and quality improvement.

### Education and professional development

Multiple healthcare education and professional-development programs for improving patient safety are currently offered by various organizations, including the CPSI, provincial organizations, professional associations, and academic centers. The CPSI, in partnership with the Canadian Healthcare Association, offers a Patient Safety Officer Course [19]. This four-day program is a comprehensive patient safety course designed for healthcare professionals and leaders who have formal responsibility for disseminating patient safety principles and programs throughout their organizations. The CPSI also provides the Patient Safety Education Project, [20] a partnership with Northwestern University, Chicago, USA. The 2 1/2 day course, built on the training team model, focuses on core patient safety content and teaching approaches to effectively drive patient safety improvement in healthcare organizations. Training programs in disclosure of adverse events to patients are offered by the Canadian Medical Protective Association CMPA [21] and the Institute for Healthcare Communication-Canada [22]. In Ontario, the Ontario Hospital Association offers a three-day patient safety course where patient safety principles, practices, and tools are presented to assist health care providers and organizations in developing patient safety programs [23]. The University of Toronto Centre for Patient Safety [24] offers a certificate course in patient safety. In twelve four-hour sessions over six months attendees learn how to plan, implement, and evaluate patient safety and quality improvement projects. Queen's University, Kingston, Ontario, is preparing to launch the first Canadian master's program in patient safety, quality, and risk. Many other education programs are currently available in most Canadian provinces.

### Information and communication

Work is currently being done on a shared Canadian electronic medical record system [25].

Major effort has been made in Canada, as in many other countries, to improve patient safety; however, evidence suggests that improvement occurs gradually, at times quite slowly [26]. Nevertheless, numerous reports have been published by Canadian organizations on improvement in clinical outcomes, such as ventilator-associated pneumonia, central line- and other hospital-acquired infections, secondary to implementation of strategies and techniques promoted by the CPSI [14]. Possibly, different approaches to improvement, such as emphasis on human factors engineering and cognitive psychology to overcome the human limitations in processing data and coping with the ever-increasing demands in health care, are required. Clearly, strong support systems are required to investigate alternatives, implement effective interventions, and ensure their reliable execution. Furthermore, the guiding principle of "no blame", which is the cornerstone of the systems approach to safety improvement, [27] has been questioned and a stronger role for accountability - both organizational and personal - has been proposed as an important mechanism to effect change [28]. While the validity of this view is highly debatable, [29] it is well accepted that accountability can be demanded only after profound improvement processes have been implemented in an organization.

## The Israeli perspective

After the 1966 National Academy of Sciences report, in Israel, "Doctors for Trauma", a group of IDF physicians, observed that the Israeli reality was similar. They claimed that trauma-related mortality would decrease by 40% if hospitals opened trauma units and if Magen David Adom (the Israeli national emergency medical services) would join the "trauma system". The Ministry of Transportation, not without resistance, eventually supported the required services. As predicted, trauma-related mortality decreased substantially [30].

**Figure 1:**
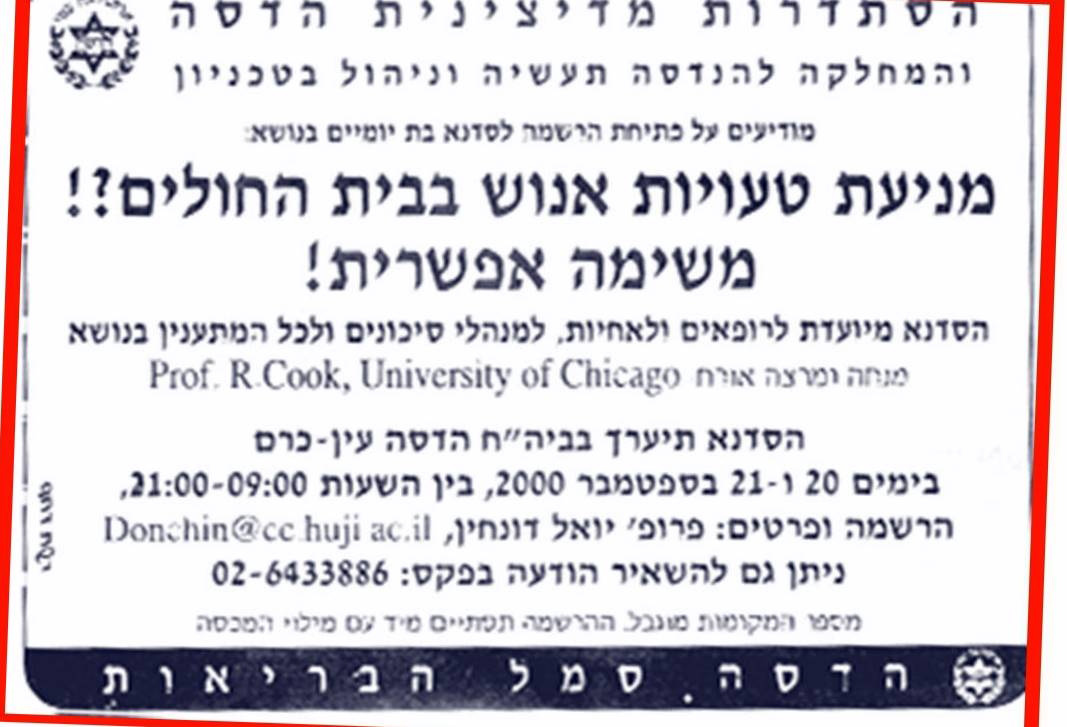
**Invitation for the first conference on medical safety in Israel, September 2000**. "Preventing human errors in the hospital?! A possible mission!"

The beginning of patient safety activity in Israel is documented in *The Patient Safety Handbook*[31]. Without any support, a group of physicians and Human Factors engineers carried out one of the first studies on the nature and causes of human error in the intensive care unit [32]. The chapter "How We Started Patient Safety in Israel: Without a Budget but with Enthusiasm" describes how hard it was at the time to publish the study's findings, a rate of 1.7 errors per patient per day. It took years to get partial recognition that we were dealing with an epidemic that is not limited to the North American healthcare system. Hadassah Medical Center joined the Center for Safety at Work and Human Factors Engineering (Technion, Haifa) and together they organized the first conference on medical safety in Israel titled: Avoiding Human Error in the Hospital: Mission Possible?! (see Figure 1). Dr. Richard Cook from the University of Chicago was the keynote speaker and delivered the message that this epidemic is treatable. He took a picture of the 30 participants and told them that this was the nucleus of the future safety movement. Over a decade later, it seems that Dr. Cook was right: all of the participants are active in different domains of the patient safety movement.

Unfortunately, the Israeli government has not taken a leading role in this mission. In spite of continuous demands and requests by a group of researchers from the Technion and Hadassah Medical Center to the Ministry of Health to adopt the American and Canadian approach to improving medical safety, including the submission of a comprehensive plan to reduce medical errors on a national level, a transformative change has not happened yet.

The Israel Ministry of Health has been investigating errors reported by law, as well as patient complaints and occasionally work processes in various healthcare settings. Patient safety alerts have been disseminated to patient safety officers at relevant medical institutions. Working under the confidentiality laws provided by the Patients' Bill of Rights 22.a, patient safety aggregated information, and the results of investigations, alongside and occasionally with the aid of investigators from the Technion, have been transformed into government issued guidelines and directives aimed at increasing patient safety. However, implementation and enforcement of these regulatory guidelines has traditionally been left to hospital directors, as part of the general belief within the ministry that its role should be one of governance and guidance and that active management should be left to individual hospital directors.

Throughout the years, the ministry has issued several required organizational practices (ROP), such as a double check before the administration of blood products, aiming to improve patient safety. Adhering to such ROPs can certainly decrease the rate of adverse events. However, a reliable process has to be designed and human resources have to be considered before a new directive is given. Furthermore, ongoing evaluation of compliance and clinical outcomes has to take place to inform the effectiveness of any intervention. This approach has not been taken. Indeed, similar to the literature in the area, investigations of several critical incidents have revealed that failures were commonly attributed to problems in the system, and only rarely to personal negligence [personal communication, Yoel Donchin, 2011].

The company providing malpractice insurance to Israeli physicians has been requested to provide clinicians with basic knowledge on human factors engineering to reduce error. As this is a private, for-profit company, it is not surprising that the request was rejected.

A call to implement safety programs has come from Clalit Health Services and from Hadassah Hospital, later from Maccabi Healthcare Services and other private healthcare groups. Understanding that a proactive approach, not just looking back at reported incidents, was needed, weak links in the system have been examined. Currently, at Hadassah two full-time physicians are assigned as safety officers. The Israel Medical Association (IMA), although still represented by lawyers, not by safety specialists, eventually accepted the need to look at safety not just from the union perspective but as a problem that must be addressed.

A range of activities to address different aspects of patient safety has been initiated sporadically by various organizations in Israel. The Quality Assurance department of the Ministry of Health has been leading quality audits nationwide, after developing a peer reviewed system that allows institutions to build and compare quality and safety indicators in a national, equal, inexpensive, and non-threatening methodology. In addition, the government has led the quality assessment program in a primary care clinics project [33]. Efforts in monitoring quality of healthcare services have been also made by the Israel Medical Association and by some of the sick funds. The Clalit Health Services have fully accredited six of their general hospitals with the International Joint Commission; the rest of their hospitals are currently in the process and plan to complete accreditation within the next two years. Five government hospitals are now in the process of preparing for accreditation review, and the next phase is to complete the process at all government-owned hospitals. Education and training for healthcare providers has been increasing and courses are currently offered by Clalit Health Services (school for patient safety), the Israeli Forum for Patient Safety (course in patient safety), all medical schools, as well as the Center for Medical Simulation at the Sheba Medical Center.

In fact, given the much lower level of resources available for patient safety in Israel than in Canada, it is noteworthy that so many activities have been undertaken by a wide range of Israeli governmental and non-governmental organizations. At the same time it is clear that with more resources and better coordination more could be accomplished.

## Developing patient safety capacity in Israel

There is a need to improve patient safety in the Israeli healthcare system. The incidence of adverse events and their impact on patients in Israel is unknown, simply because research and data are lacking. However, there is no reason to assume that Israeli reality is different from that in other western countries: roughly one in every 15 patients admitted to a hospital suffers an adverse event.

The question is therefore as follows: what steps should be taken at the national level to establish an organized approach to improving patient safety? To answer this question, the following points should be considered:

1. Evidence of demonstration/proof of concept. The capacity to investigate, intervene, and teach patient safety has been well demonstrated in Israel by several pockets of excellence. Therefore, a comprehensive program in patient safety improvement could be informed by and seamlessly tied into these already existing activities.

2. Availability of core content. The Patient Safety Center in Hadassah Medical Center, for example, has been implementing the full core content of such a program, including research, education, and an active service dedicated to patient safety improvement. Their approach is based on the body of knowledge of cognitive psychology and human behavior. Similar approaches will need to be disseminated throughout the healthcare system.

3. Education dissemination. Currently, patient safety is a core subject only in some medical and nursing schools in Israel. Sporadic teaching occurs in centers where there are teaching personnel with specific interest in the topic, as well as by some organizations, as mentioned above. A focus on education will be crucial to make progress.

4. Culture alignment; public media. The Israeli public has been exposed occasionally to discussion of adverse events and issues related to patient safety. For example, overcrowding of patients on hospital wards and poor working conditions of healthcare workers were recently discussed in Israeli media [34]. Therefore, the need for improving patient safety is likely to be well accepted by the public.

5. Specialty standing; provider accreditation. Making patient safety an independent specialty, practiced by various healthcare professionals, would likely establish the importance of the field and generate further education, research, and other activities.

6. Service capacity, institutional accreditation. General accreditation processes have been gradually introduced to healthcare organizations in Israel. Means to enforce compliance with education, measurement, and performance, as they relate to patient safety practices, should be integrated into the already existing mechanisms and established wherever they do not currently exist.

7. Research. There is currently no coordinated patient safety research strategy in Israel. This would be a major target for improvement.

8. Policy & financing; access and service integration; utilization; quality sustainment. Since patient safety services are currently not regulated in Israel, these important aspects will need to be addressed as part of a national program.

## Proposed immediate next steps for Israel

There are important differences between the Israeli and Canadian healthcare systems. For example, medico-legal insurance is provided in Israel by a private company, as opposed to not-for-profit organizations in Canada. In addition, Canadian healthcare is provided almost exclusively by the provincial governments, whereas in Israel several providers exist, including public, semi-private, and private systems. Finally, the research budget is quite limited in Israel compared with Canada. Features of the Israeli healthcare system will need to be considered when designing a system to create patient safety capacity in Israel.

Therefore, we suggest that the following steps be taken immediately:

1. Establish an Israeli patient safety institute (IPSI). This government-funded, not-for-profit organization will be the major driving force for coordinated efforts to address all aspects of patient safety improvement, including legislation and regulation, measurement and evaluation, research, information and communication, and education.

2. All major providers of healthcare services will be required to establish and support patient safety departments. These bodies, in collaboration with the IPSI, will set priorities, intervene as appropriate, and coordinate education and support to all workers and their patients.

3. Adequate budget will be allocated to research activities to inform decision making and investments in the area.

4. Education of patient safety will be mandated at all levels of the healthcare system, including medical and nursing schools and healthcare organizations. Existing educational programs such as the Patient Safety Education Project [19] could be brought to Israel with a relatively small budget and combined with locally existing expertise and infrastructure.

## Conclusion

Injuries to patients by the healthcare system are common and their impact on individuals and systems is considerable. Over the last decade, extensive efforts have been made throughout the world to improve patient safety. Whereas the road to improvement is long and achievements are slow to materialize, the extent of activities must be coordinated and organized at the national level. Such a process has not yet begun in Israel. While some basic concepts and expertise already exist in Israel, there is a considerable gap that needs to be filled. We suggest several steps that should be taken immediately to narrow the performance gap and improve safety for patients in Israel. Learning from the experience of other countries and using already existing resources would save time and money.

## Abbreviations

ACE: Angiotensin Converting Enzyme; AMI: Acute Myocardial Infarction; CEO: Chief Executive Officer; CIHI: Canadian Institute for Health Information; CMPA: Canadian Medical Protective Association; CPSI: Canadian Patient Safety Institute; EMS: Emergency Medical Services; HSMR: Hospital Standardized Mortality Ratio; IHI: Institute for Healthcare Improvement; IMA: Israel Medical Association; IPSI: Israel Patient Safety Institute; ROP: Required Organizational Practice; U.K.: United Kingdom; U.S.: United States.

## Competing interests

The authors declare that they have no competing interests.

## Authors' contributions

RI and YD conceived and drafted this manuscript. All authors read and approved the final manuscript.

## Authors' information

Roy Ilan, MD, MSc is an Assistant Professor at Queen's University, Canada and is a critical care and internal medicine physician at Kingston General Hospital. He serves as Faculty at the Master's program in Healthcare Quality at Queen's University, and as a Master Facilitator for the Canadian Patient Safety Institute's Patient Safety Education Program.

Yoel Donchin is a retired anesthesiologist and head of the Hadassah University Hospital's patient safety unit. He is a member of the Technion's Center for Safety at Work and Human Factors Engineering, in Haifa, Israel. His book (with Daniel Gopher), entitled "Around the patient bed - Human factors and safety in health care" (Hebrew) was recently published by Carta http://www.carta.co.il/carta/products/01378.html, publishing house, Jerusalem.
